# Protective and therapeutic potentials of HDL and ApoA1 in COVID-19 elderly and chronic illness patients

**DOI:** 10.1186/s42269-022-00886-x

**Published:** 2022-07-28

**Authors:** Mohamed Aly AbdelHafez

**Affiliations:** Department of Medical Biochemistry and Molecular Biology, School of Medicine, Cairo University, Kasr AlAiny, Al-Manyal, Cairo, Cairo 11562 Egypt

**Keywords:** COVID-19, HDL-Apo-lipoprotein A1, Elderly subjects, Chronic illness, Hemoglobin-haptoglobin-hemopexin, Apo-A1 mimetic peptide D-4F, ω-3 polyunsaturated fatty acids, *Saussurea lappa* (costus), ApoM-sphingosine-1-phosphate

## Abstract

**Background:**

Severe acute respiratory syndrome coronavirus 2 (SARS-CoV-2) is responsible for coronavirus disease-2019 (COVID-19). Elderly subjects, obese, and patients with chronic illnesses, are the most affected group. HDL has pleiotropic physiological functions that are affected with alteration(s) in its structure.

**Main text:**

Inflammation whether septic, immune, or other affects HDL structure and function. COVID-19 is associated with systemic immune-inflammation due to cytokine surge. Viral interaction with erythrocytes and hemoglobin-related compounds (may cause anemia and hypoxia) and other factors may affect HDL function. Trials have been conducted to resume HDL functions using peptide preparation, nutritional, and herbal elements.

**Conclusions:**

In this review article, I’ll discuss the use of reconstituted HDL (rHDL), Apo-A1 mimetic peptide D-4F, ω-3 polyunsaturated fatty acids, and the powdered roots and/or extract of *Saussurea lappa* (costus) to avoid comorbidity and mortality of COVID-19 in patients with chronic illness or elderly-age mortality.

## Background

HDL has multiple physiological functions; i.e. reverse cholesterol transport, anti-inflammatory, anti-thrombotic, anti-oxidative, anti-apoptotic in addition to protection of vascular endothelium against damage and leakage of small particles like LDL to pass through. Apo-lipoprotein A1 (Apo-A1) is the main apo-lipoprotein component and plays an axial role in HDL function. Alterations of HDL disturb its functions. Elderly subjects, obese patiens, and patients with chronic illness such as diabetes, cardiovascular disease (CVD) or chronic inflammatory diseases have bad comorbidity among COVID19 patients. Inflammation affect HDL structure and function. COVID-19 causes systemic immune-inflammation due to cytokine explosion, viral interaction with erythrocytes and its hemoglobin content and other factors influencing HDL function. Clinical and experimental trials suggest that resuming the HDL function by administration of reconstituted HDL, Apo-A1 mimetic peptide D-4F, ω-3 polyunsaturated fatty acids, and/or the powdered roots of Saussurea costus ameliorates the clinical outcome. We recommend application of these to regain HDL protective function in the fore-mentioned cases.

## Main text

### HDL structure and function

#### Reverse cholesterol transport (RCT)

Apolipoprotein Apo A-1 interacts with ATP-binding cassette-1 (ABCA1) in various cell types (hepatocytes, enterocytes, and macrophages). Cholesterol and phospholipids are combined in this interaction to form nascent HDL particles (pre-HDL, or HDL3), which are discoid HDL. Other apolipoproteins may be added, except for Apo-B.

Cholesterol is esterified with unsaturated fatty acid of lethicin to form a mature molecule of HDL (α-HDL or HDL2); catalyzed by lecithin-cholesterol acyltransferase (LCAT). Cholesterol ester is transferred to Apo B-100-containing lipoproteins, especially to the low-density lipoprotein (LDL), in exchange for triacylglycerol to form spheroid HDL. Apo B-100-containing lipoprotein binds to LDL-receptor on the hepatocyte surface and undergoes endocytosis to eliminate its cholesterol content. Mature HDL interacts with *scavenger receptor-B1* (SR-B1) in the liver, this allows the transfer of its cholesterol content as well as triacylglycerol (TG). By release of TG, the remained HDL molecule, the pre-β HDL (HDL3) circulates and repeat the RCT process (Steck and Lange 2010).

In addition to Apo A-1, HDL particles contain ApoM, enzymes involved in antioxidative mechanisms such as paraoxonase-1, lecithin-cholesterol acyltransferase, and diverse lipid species, including cholesterol esters, triglycerides, phospholipids, and bioactive sphingolipids such as sphingosine 1-phosphate (S1P) (Nofer et al. 2004). In the systemic circulation, about 60% of plasma S1P is bound to apolipoprotein M (apoM) (Kurano and Yatomi 2018). S1P signals through specific G proteins. It inhibits vascular permeability and is required for vascular development as well as immune cell trafficking (Liu et al. 2000). ApoM is involved in an anti-inflammatory signalling complex that inhibits NF-B-dependent inflammatory pathways. The NF-κB pathway is a key inflammatory signaling pathway induced by TNFα (Galvani et al. 2015).

### Disturbed HDL protective function against oxidation and inflammation

HDL anti-oxidant function is attributed to paraoxonase-1 (PON-1) enzyme (James et al. 2010). Variation in paraoxonase-1 activity is recorded in atherosclerosis and other inflammatory states (Soran et al. 2009).

Exposure of HDL to oxidation stress, e.g. at sites of inflammation, diminishes Apo A1 activity (Zheng et al. 2004), and the LCAT biological function (Shao et al. 2008) resulting in disturbed HDL turnover.

Systemic inflammation can also contribute to the observed hypoxia-mediated HDL impaired activity (Biolo et al. 2018). Owing to their small particle size, LDL get access into extracellular fluid to be in direct contact with tissue cells. Reactive oxygen species (ROS) are generated in tissues owing to cycloxogenase, lipooxygenase, myeloperoxidase, and/or NADPH oxidase exaggerated activity during inflammatory process. LDL is oxidized into oxy-LDL. HDL3 (pre-β-HDL), protect LDL from oxidative damage by free radicals (Kontush and Chapman 2010). Native Apo A-I inhibits the proinflammatory effect of oxidized lipids (Navab et al. 2000). At sites of inflammation, myeloperoxidase bound to HDL, catalyzes its oxidation to a proinflammatory particle (Undurti et al. 2009) as evidenced by elevation of CRP (Corsetti et al. 2010). Fatty acids moieties of phospholipids in plasma membranes and lipoproteins are oxidized to lipid hydroperoxides. Lipid hydroperoxides migrate from the surface of LDL to be transfered to the liver via SR-B1 and/or to be carried by HDLs (Bowry et al. 1992). The imbalance in the antioxidant system causes oxHDL modification. Oxidized lipids enhance circulating macrophages to release pro‐inflammatory cytokines (Van Lenten et al. 1995). Cytokines decrease synthesis and/or secretion of lipoproteins including Apo-AI which are steadily decreased as the disease progress to critically ill state.

Inflamattory cytokines increase the activity of secretory phospholipase A2 (sPLA2) and endothelial cell lipase, enzymes that metabolize key HDL constituents (Filippas-Ntekouanet et al. 2017). Insulin resistance reduces lipoprotein lipase (LPL) activity (Popko et al. 2010). Clearance of triglyceride content of chylomicrons and VLDL is blunted. Transfer of cholesteryl-esters from HDL to ApoB-lipoprotein series is inhibited. Hence, cholesterol efflux from peripheral tissues is retarded. Altered HDL-apolipoprotein structure inhibits release and activation of hepatic lipase with elevation of plasma TG (Chatterjee and Sparks 2011). Hypertriglyceridemia is among the factors potentiating cardiometabolic risk (Moriyama and Takahashi 2016). Plasma levels of apoM in diabetics is reduced as compared with euglycemic control subjects. ApoA-I, apoM, and S1P in insulin resistance are decreased (Kurano et al. 2020).

Serum amyloid A (SAA) may displace ApoA-I in HDL particles, leading to increased catabolism of HDL (McEneny et al. 2016). This is an additional factor responsible for shifting the function of HDL from a vasoprotective to a pro-atherosclerotic lipoprotein (Zewinger et al. 2015). SAA plasma levels are progressively elevated with COVID‐19 disease severity, so a prognosis of the disease could be evaluated (Li et al. 2020).

Low HDL-C levels consist a poor prognostic factor for COVID-19 severity (Agouridis et al. 2021). High HDL-C levels were associated with a lower risk of hospitalization due to COVID19 infection (Lassale et al. 2021). Serum ApoA1 is reduced with the progress of illness in COVID-19 patients (Yang et al. 2020).

### Interaction of hemoglobin and related molecules with HDL

Normally, HDL is bound at low levels of hemoglobin associated to its Apo A-I fraction. Hemoglobin / HDL is increased in inflammatory states. Haptoglobin is a protein synthesized by the liver to bind circulating plasma free hemoglobin and facilitate its association to HDL (Watanabe et al. 2009). Hemopexin, a protein involved in iron transport, is identified among HDL proteins. The interaction of hemoglobin, haptoglobin, and hemopexin with HDL is positively correlated with the proinflammatory properties of HDL during systemic inflammation so that it reduces the activity of Apo A1 (Spagnuolo et al. 2005). Accordingly, increased HDL linked hemoglobin may contribute for higher levels of lipid hydroperoxides besides acquiring proinflammatory characteristics (Watanabe et al. 2007).

### Interaction of SARS-CoV-2 infection with erythrocytes

The viral effect on heme metabolism is due to binding of viral surface glycoprotein with the beta-chains of hemoglobin (Wenzhong and Hualan. 2020a) resulting in hemoglobin denaturation (Wenzhong and Hualan. 2020b). Hence, SARS-CoV-2 may induce hemolysis. During the course of the disease, anemia progresses and hemoglobin-related pathology also progresses. Hemoglobin/iron interrelation may result in multi-organ disorders and systemic hypoxia.

An additional receptor, CD147, was identified besides angiotensin converting enzyme 2 (ACE2) on erythrocytes and other cells (Wang et al. 2020). CD147 may be the routes through which the virus get access into the erythrocytes, bone marrow immature cells (Ulrich and Pillat 2020). Cardiac pericytes, vascular smooth muscles and probably vascular endothelium may be invaded by the virus through CD147 (Robinson et al. 2020).

Hypoxia, which develops as a sequence of erythrocytic hemoglobin and cardiovascular affection, may upregulate CD147 expression. Obese and diabetics overexpress CD147 receptors in erythrocytes (Radzikowska et al. 2020). This finding may add an explanation of comorbidity of COVID-19 in obese and diabetic subjects.

### Dysorganisation of iron metabolism in SARS-CoV-2 infection

Infection with SARS-CoV-2 has hepcidin-like activity, probably mediated by IL-6 induced gene expression. Hepcidin facilitates iron accumulation in cells via downregulation of ferroportin (Means Jr 2022). The latter is the key transporter of iron outside the cells. Plasma iron is decreased (hypoferremia) with higher ferritin concentration (hyperferritinemia) (Nemeth et al. 2004). Erythropoiesis becomes inadequate resulting in anemia of inflammation (Ganz and Nemeth 2011). Since blood S1P, bound to HDL-apoM, is linked to number of erythrocytes (Hänel et al. 2007), anemia is associated with decrease in plasma S1P and the circulating S1P is prognostic and predictive biomarker in COVID-19 patients (Marfia et al. 2021). Serum iron ensues contributing to hypoferremia (Drakesmith and Prentice 2012). Increased intracellular iron content potentiates SARS-CoV-2 replication in affected cells (Kalyanaraman 2020). Early in COVID-19 patients, cell iron overload is tolerated without apparent hypoxia. According to Fenton reaction, iron is a potent pro-oxidant; that increases reactive oxygen species (ROS) creating oxidation stress state. ROS activates nuclear factor-kappa B kinase (Lingappan 2018); potentiating the inflammatory response. Furthermore, increased intracellular iron upregulates the expression of inflammatory factors such as IL-6, IL-8, and TNF-α, which in turn, aggravate cytokine surge (Girelli et al. 2021). Concurrently, anemia caused by lower hemoglobin level and hyperferritinemia, besides hemoglobinopathy (caused by SARS-Cov-2 binding to β-chain of hemoglobin) are risk factors contributing to bad comorbidity of mild case to critically-ill condition (Zhou et al. 2020).

### Effect of hypoxia

Hypoxia changes lipoprotein pattern in an atherogenic direction by lowering HDL-C particularly, HDL2-C (Biolo et al. 2018).

Hypoxia induces release of hypoxia inducible factor-1 (HIF-1). Hypoxia and HIF-1α can either stimulate or inhibit cytokine-mediated inflammatory response. The stimulation process depends on upregulation of vascular endothelial growth factor (VEGF) from vascular endothelium. VEGF is among factors that contributes for pathogenicity of severe COVID-19 (Teuwen et al. 2020), by recruiting circulating neutrophils, macrophages, mast cells, and dendritic cells. These cells as well as vascular endothelial cells release ROS and proteases besides cytokines, adhesive molecules, and chemoattractants (monocyte chemo-attractant protein-1, interleukin-1 β, and chemical cytokine-2). VEGF increases cytokine expression and vascular permeability (Shibuya 2011). These factors are responsible for progress of the pathological critical–ill status.

Hypoxia, in addition, triggers mitochondrial dysorganization, altering the mitochondrial membrane permeability so that ROS generation increases and ATP generation is depressed. Post-inflammatory cell damage releases its content of ATP into the extracellular matrix that increases tissue content of adenosine. Adenosine is an anti-inflammatory factor (Rajasundaram 2018). On the other hand, hypoxia induced HIF-1α switches metabolism to anerobic glycolysis which promotes the accumulation of adenosine in an attempt to combat inflammation (Sitkovsky et al. 2014). This might be a protective measure in the course of the disease.

## Resolving mediators in COVID-19

Persistence and continuity of the inflammatory response is driven by the “cytokine storm,” which is triggered by release of the pro‐inflammatory cytokines throughout the course of the disease. Other influencing factors are oxidation stress, altered lipoprotein structure and function, hypoxia, and changes in hemoglobin-interacting compounds.

Recovery from the disease condition is not a passive mechanism that depends just on elimination of these factors. Rather, it is an active process initiated by what are so-called *specialized pro-resolving mediators (SPMs)*.These are lipid mediators derived from ω3-polyunsturated fatty acids linked to phospholipids of plasma membrane of macrophages and neutrophils as well as lipoprotein phospholipids (Serhan et al. 2008). There are two series of SPMs, E-series derived from eicosapentaenoic acid (EPA); resolvins, and D‐series derived from docosahexaenoic acid (DHA); protectins and maresins. ω3-Fatty acids (EPA and DHA) compete with ω6-polyunsaturated fatty acid; arachidonic acid (the precursor of proinflammatory mediators) for cyclooxygenase and lipooxygenase enzymes, thus inhibiting biosynthesis of prostaglandins, leukotrienes, thromboxaneA2, and lipoxins. SPMs potentiate phagocytosis of apoptotic cells and cell debris left in the inflammatory process by phagocytic cells (the so-called “efferocytosis”) (Serhan et al. 2008). This process pivots on recovery from the inflammatory state (El Kebir et al. 2012). It has been reported that DHA‐derived protectin D1, is a suppressor of influenza virus replication and regresses its severe symptoms (Morita et al. 2013). Combination of acetyl salicylic acid with EPA and/or DHA ameliorate severe respiratory symptoms and coagulopathy features in patients with COVID-19 (Das 2020). Obese subjects have SPM deficits. This may be responsible for the worse morbidity of their COVID‐19 course owing to lack of these resolution factors (Pal et al. 2020). EPA- and DHA-linked HDL improves it antioxidant and enzyme functions (Cartolano et al 2022).

## *Saussurea lappa* (costus); phytotherapeutic agent

*Saussurea lappa* (costus) is a plant well-known about 2,500 years ago and used traditionally in the Indian and Arab systems of medicine. Mostly the essential root oil and root powder were used for the medicinal purposes (Nikhat and Fazil 2020). *Saussurea lappa* (SL) has anti-inflammatory activity as documented by reduction of RNA expression of inflammatory cytokines: TNF-α, GM-CSF and IL1β and metalloprotease-9 activity (Lammari et al. 2021). It inhibits protein and mRNA expression of interleukin-1b (IL-1b) as well as inhibition of phosphorylation of mitogen activated protein kinases (MAPK) (Kang et al. 2004). It suppresses expression of hepatitis B surface antigen (HBsAg) in human hepatoma Hep3B cells in a dose-dependent manner (Chen et al. 1995) and ameliorates chronic hepatitis B (Ansari et al. 2018) suggesting anti-viral activity. Besides, SL components have anti-diabetic, and anti-lipidemic activities (Gomaa et al 2020) raising the possibility of improving HDL levels and functions. It has been reported to be effective in prevention and treatment of COVID-19 by a research group in Upper Egypt. Their findings were published in a local Periodical by Saif-Al-Islam M, and colleagues (Sohag Med J. 2020; 24(3): 6–1).

## Targeting therapeutic and preventive potentials of HDL

It is essentially important for healthy elderly who potentially have low levels of healthy functioning HDL-ApA1 to protect them from caching this life-threatening disease; covid-19. Elderly subjects with chronic Illnesses (diabetes mellitus, obesity, chronic CVD, obstructive airway diseases, hematological diseases, autoimmune diseases), or immune-compromised subjects are also candidates of applying preventive measures.

Considering the functions of HDL and its composition that is altered during acute-phase response; the oxidative status at a site of inflammation modifies HDL proteins, making them proinflammatory besides loss of its anti-oxidant property. The increased association of HDL-hemoglobin-haptoglobin-hemopexin potentiates these changes in addition to inflammatory anemia and increasing hypoxia. HDLs from patients with COVID-19 are less protective in endothelial cells stimulated by TNFα. In these conditions, HDL inhibition of apoptosis was blunted in COVID-19 (Begue et al. 2021).

Reconstituted HDL (rHDL) containing pre-β HDL (HDL3) supplementation, could be a preventive and/or therapeutic target against COVID-19. Difficulties in preparation and supply are a limitation to achieve this purpose. Some investigators have been used Apo A-I mimetic peptide D-4F. D-4F is N-terminal acetylated and C-terminal amidated apoA-I mimetic peptide, Ac-D-W-F-K-A-F-Y-D-K-V-A-E-K-F-K-E-A-F-NH_2_ that is synthesized from 18 D-amino acids (Navab et al. 2005). D-4F potentiates the anti-inflammatory function of HDL and recover the reverse cholesterol transport (Bloedon et al. 2008). It recovers anti-oxidant function of HDL by increasing the PON-1 activity in mice leading to decreased plasma levels of oxidized fatty acids (Navab et al. 2004). Many physiological functions of HDL were regained in experimental animals by using D-4F peptide (Dai et al. 2010; Smythies et al. 2010).

Inclusion of ω3-fatty acids EPA and DHA (as precursors of resolvins, protectins and maresins), in the therapeutic protocol of COVID-19 may be valuable in suppressing the inflammatory process. Their use early in the disease is recommended to suppress the inflammatory reactions and to eliminate the tissue debris left by necrobiosis (Sorokin et al. 2020). They, in addition, enhance viral clearance through interrupting the viral enveloped protein formation (Pal et al. 2020). Arachidonic acid being the precursor of pro-inflammatory prostanoids; its use is not recommended in this respect.

## Conclusions

Apo-A1 mimetic peptide D-4F, ω-3 polyunsaturated fatty acids, and the powdered roots and/or extract of *Saussurea lappa* (costus) are suggested means for resuming HDL-ApA1 physiological activity aiming at protecting elderly and chronically ill patients and reducing the morbidity of COVID-19. More investigations may be recommended for safety and efficacy.

## Data Availability

Not applicable.

## Abbreviations

α-HDL-C: Alpha high density lipoprotein-cholesterol
ABCA1: ATP-binding cassette-1
ABCG1: ATP-binding cassette-G1
Apo-A1: Apo-lipoprotein A1
Apo-M: Apolipoprotein M
CETP: Cholesteryl ester transfer protein
COVID-19: Coronavirus disease-2019
CRP: C-reactive protein
CVD: Cardiovascular disease
D-4F: Apo A-I mimetic peptide made of 18 D-amino acids of which 4 residues of phenylalanine.
DHA: Docosahexaenoic acid
EPA: Eicosapentaenoic acid
HDL-ApA1: High-density apolipoprotein A1
HIF-1: Hypoxia inducible factor-1
IL‐6: Interleukin-6
LCAT: Lecithin-cholesterol acyltransferase
PON-1: Paraoxonase-1
RCT: Reverse cholesterol transport
rHDL: Reconstituted HDL
ROS: Reactive oxygen species
S-1-P: Sphingosine-1-phosphate
SAA: Serum amyloid A
SARS-CoV-2: Severe acute respiratory syndrome coronavirus-2
sPLA2: Secretory phospholipase A2
SL: *Saussurea lappa*
SPMs: Specialized pro-resolving mediators
SR-B1: Scavenger receptors B1
TG: Triacylglycerol
TLR: Toll-like receptor
TNF‐α: Tumor necrosis factor‐alpha
VEGF: Vascular endothelial growth factor
